# Narrative review and call to action on reporting and representation in orthobiologics research for knee osteoarthritis

**DOI:** 10.1002/pmrj.13214

**Published:** 2024-07-06

**Authors:** Alexander Sin, William Hollabaugh, Lauren Porras

**Affiliations:** ^1^ Division of Sports Medicine, Department of Orthopaedic Surgery Vanderbilt University Medical Center Nashville Tennessee USA

## Abstract

Osteoarthritis affects a significant portion of U.S. adults, and knee osteoarthritis contributes to 80% of disease burden. Previous data have shown that non‐White patient populations often report worse symptoms and less favorable outcomes following arthroplasty, a definitive treatment for knee osteoarthritis. There is a lack of demographics data on race/ethnicity, as well as socioeconomic status (SES) and social determinants of health (SDOH), in knee osteoarthritis treatment guidelines and knee arthroplasty research. In addition, there is underrepresentation of non‐White patient populations in the existing treatment guidelines for knee osteoarthritis. Over the past decade, orthobiologics have emerged as an alternative to surgical intervention. Our hypothesis is that there would be a similar lack of reporting of demographics data and underrepresentation of non‐White populations in studies pertaining to orthobiologics, including evaluating differences in outcomes. This study reviewed U.S.‐based research in orthobiologics as a treatment option for knee osteoarthritis. We identified a lack of demographics reporting in terms of race/ethnicity, and none of the studies reported SES or SDOH. Non‐White populations were underrepresented; White patients contributed to 80% or more of all study populations that reported race/ethnicity. None studied the correlation between symptoms and outcome measures, and the race/ethnicity, SES, and SDOH of the patients. Based on a review of existing literature, we strongly advocate for ongoing research encompassing patients of all races/ethnicities, SES, and SDOH, and an exploration into potential variations in symptoms and outcomes among distinct population subgroups. Furthermore, SES barriers may influence health care delivery on orthobiologics for disadvantaged populations.

## INTRODUCTION

The Centers for Disease Control and Prevention (CDC) estimates that osteoarthritis affects over 32.5 million adults in the United States, with an associated treatment cost of 185.5 billion dollars per year.^1^ Knee osteoarthritis accounts for more than 80% of total disease burden and affects at least 19% of the U.S. population 45 years of age or older.^2^ There are well‐established variations in knee osteoarthritis in patients of different race/ethnicity and socioeconomic status (SES)^3^: knee osteoarthritis is 50% to 60% more prevalent in African Americans when compared to Caucasians, and a recent meta‐analysis on racial/ethnic differences in osteoarthritic pain and disability has found higher pain severity in African Americans than in Caucasians.^4^ Similarly, low education was found to be associated with a higher prevalence of radiographic knee osteoarthritis, and low education and higher community poverty rates were both found to be significantly associated with worse pain and function in the Johnston County Osteoarthritis Project.^5^, ^6^ Intersectional data have suggested that African Americans with osteoarthritis living below the poverty line may be at the greatest risk for poor pain and functional outcomes.^7^

With respect to treatment, non‐White race and lower socioeconomic background have been consistently shown to be associated with reduced access to physical therapy and lower likelihood of receiving total joint replacement.^8^ In an analysis of Medicaid enrollees, more money was spent on treating knee osteoarthritis of African American patients,^9^ whereas this group is also reported to have worse functional outcomes and more complications with total joint replacement.^8^

Research pertaining to the utility of orthobiologics has increased significantly over the past decade,^10^ and numerous studies have examined the use of platelet rich plasma (PRP),^11^, ^12^, ^13^, ^14^, ^15^, ^16^, ^17^ bone marrow aspirate concentrate (BMAC),^18^, ^19^, ^20^, ^21^, ^22^, ^23^ and microfragmented adipose tissue (MFAT)^19^, ^23^, ^24^, ^25^, ^26^ in knee osteoarthritis. The body of research thus far has suggested that these treatments are safe, and that they may be a non‐inferior alternative treatment for knee osteoarthritis. The most recent positional statement from American Medical Society for Sports Medicine (AMSSM) on regenerative medicine supported the notion that PRP injections are more effective than steroid or hyaluronic acid injections for knee osteoarthritis.^27^ More research is still needed to establish a definitive role for orthobiologics in treating knee osteoarthritis, and we are at a critical stage where research should be standardized in terms of methodology, and be intentionally inclusive with regard to participant recruitment to improve its external validity. Orthobiologics are quickly becoming commonplace in sports medicine clinics worldwide. In a recent study on orthobiologics use by sports medicine physicians, 66.1% of respondents reported using one or more types of orthobiologics, with osteoarthritis being the pathology most often treated with orthobiologics (71.6%). Furthermore, 71.6% of respondents reported increasing orthobiologics use in their practice.^10^ A recent study of a large U.S. insurance claims database showed that PRP injections quadrupled from 2010 to 2020, with a projected increase in annual usage of 66% by 2030.^28^ Cost associated with orthobiologics use is variable and dependent on factors such as clinical setting (clinic or operating room), need for anesthesia and analgesics, as well as need for image guidance.^29^ Many of these interventions are considered experimental by insurance companies, and approval of coverage is often difficult to obtain.

As multiple studies have associated non‐White racial backgrounds and lower SES with worse treatment outcomes and increased complications in total knee joint replacements, it is important to explore the efficacy and safety of orthobiologics as an alternative treatment approach across diverse populations. It is also important to ensure equitable access to orthobiologics for all populations if more definitive evidence supports orthobiologics as a safe and efficacious treatment option.

To our knowledge, no research has been performed on the patient representation in orthobiologics studies. Related studies in non‐operative and operative treatment of knee osteoarthritis often focus on reporting patient age, sex, level of sports participation (high school, college, recreational, professional), but not on the race/ethnicity or SES of the studied population. A recently published systematic review revealed that only 4.2% of 72 randomized controlled trials on total hip and/or knee arthroplasty included race, and 1.4% included ethnicity in the demographic table.^30^ A recent study analyzed the proportion of racial/ethnic groups in articles included in the 2015 American Academy of Orthopaedic Surgeons Surgical Management of Osteoarthritis of the Knee Clinical Practice Guideline, and found a significant underrepresentation of non‐White participants.^31^ This signifies that the evidence base for the surgical management of knee osteoarthritis has been constructed from studies that fail to consider race and ethnicity, which may explain why there is a systematic gap in treatment outcomes in non‐White patients.

The objective of this review is to examine the reporting of racial and ethnic characteristics, and SES such as income level and educational level, in the demographics of randomized controlled trials and retrospective cohort studies of PRP, BMAC, MFAT in the treatment of knee osteoarthritis in the United States.

## METHODS

The references used in this narrative review were obtained in July 2023 using a search of PubMed, Embase, and Web of Science online databases. Articles were retrieved using the following search terms: “Orthobiologic,” “Platelet‐Rich Plasma,” “Bone Marrow Aspirate,” “Microfragmented Adipose Tissue,” “Adipose Tissue Derivative,” “Adipose Stem Cell,” “Adipose Derived Stem Cell,” or “Mesenchymal Stem Cell,” or “Multipotent Stromal Cell,” or “Mesenchymal Stromal Cell” or “Medicinal Signaling Cell,” and combined with “Osteoarthritis, Knee,” or “Knee Osteoarthritis,” or “Osteoarthritis of the Knee,” and with “Randomized Controlled Trial,” or “Controlled Clinical Trial,” or “Trial,” or “Groups,” or “Drug Therapy,” or “Randomly,” or “Randomized,” or “Placebo,” or “Retrospective,” or “Cohort,” using the Boolean operators “AND” and “OR.” For the complete search string, please refer to Table 1.

**TABLE 1:** Search String.

Search String used in this study for Article Retrieval from PubMed, Embase, and Web of Science
(Orthobiologic*[tiab] OR “Platelet‐Rich Plasma”[Mesh] OR platelet rich plasma[tiab] OR PRP[tiab] OR bone marrow aspirate[tiab] OR BMA[tiab] OR BMAC[tiab] OR Microfragmented adipose tissue*[tiab] OR MFAT[tiab] OR adipose tissue derivative*[tiab] OR Adipose Stem Cell*[tiab] OR Adipose derived stem cell*[tiab] OR mesenchymal stem cell*[tiab] OR multipotent stromal cell*[tiab] OR mesenchymal stromal cell*[tiab] OR Medicinal signaling cell*[tiab]) AND (“Osteoarthritis, Knee”[Mesh] OR Knee Osteoarthritides[tiab] OR Knee Osteoarthritis[tiab] OR Osteoarthritis of Knee[tiab] OR Osteoarthritis of the Knee[tiab]) AND English[Language] AND ((randomized controlled trial[pt] OR controlled clinical trial[pt] OR randomized[tiab] OR placebo[tiab] OR drug therapy[sh] OR randomly[tiab] OR trial[tiab] OR groups[tiab] OR retrospective[tiab] OR cohort[tiab]) NOT (animals [mh] NOT humans [mh]))

The scope of articles examined will be limited to U.S.‐based studies for two reasons. First, most studies that examined the associations between race, ethnicity, and SES and the access to and outcome of knee osteoarthritis treatments were performed on U.S. populations. By limiting the scope of our study, we can better correlate our findings with pre‐existing studies to detect similar underrepresentation, if present. It is also more practical to directly compare the racial/ethnic representation of the U.S.‐based studies to the racial/ethnic ratios in the United States. Second, clinical trials present an opportunity to improve access by enrolling patients who would otherwise be unable to afford these procedures. Orthobiologic procedures, especially PRP, is often more affordable and sometimes covered by national medical systems or medical insurance in European countries.^32^ In the United States, these procedures are generally not covered by insurance and out‐of‐pocket costs can be incurred that will likely limit access for patients of lower SES. By limiting the scope to U.S.‐based studies, this study can better detect if clinical trials improved access for patients who would otherwise be unable to access these treatments due to their associated costs.

## RESULTS

**FIGURE 1:**
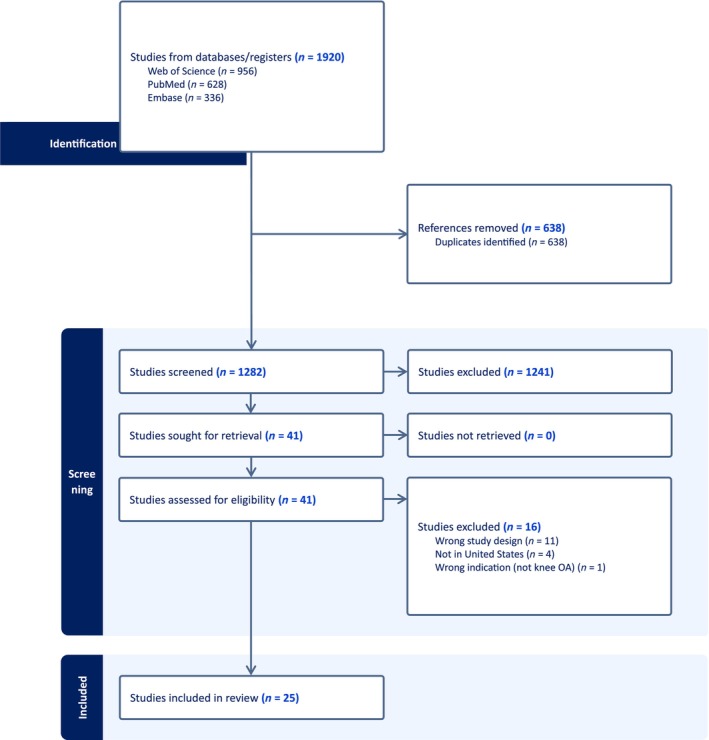
PRISMA diagram of the narrative review process.

Through our formal literature search, 1920 articles were identified. Six hundred thirty‐eight articles were found to be duplicates and were removed. Articles were then screened based on a priori established inclusion and exclusion criteria (Table 2). Our inclusion criteria included retrospective cohorts, prospective cohorts, and randomized controlled trials in English language that studied PRP, BMAC, or MFAT in the context of knee osteoarthritis. Studies that were not in English or that were performed outside of the United States were excluded. One thousand two hundred forty‐one studies were excluded from abstract screening, yielding 41 articles that underwent full‐text review, which resulted in the final 25 articles^18^, ^23^, ^33^, ^34^, ^35^, ^36^, ^37^, ^38^, ^39^, ^40^, ^41^, ^42^, ^43^, ^44^, ^45^, ^46^, ^47^, ^48^, ^49^, ^50^, ^51^, ^52^, ^53^, ^54^, ^55^ that were included in this narrative review. Please refer to Figure 1 for the Preferred Reporting Items for Systematic reviews and Meta‐Analyses (PRISMA) diagram. Twenty‐two of the final 25 articles included in the analysis were prospective,^18^, ^33^, ^34^, ^36^, ^37^, ^38^, ^39^, ^40^, ^41^, ^42^, ^43^, ^44^, ^45^, ^46^, ^47^, ^48^, ^49^, ^50^, ^51^, ^52^, ^53^, ^55^ and 11 of the 22 prospective studies were randomized controlled trials.^37^, ^38^, ^40^, ^43^, ^44^, ^45^, ^49^, ^50^, ^52^, ^53^, ^55^

**TABLE 2:** Inclusion and Exclusion Criteria.

Inclusion Criteria
PRP, OR BMAC, OR MFAT as one or more treatment arms Knee osteoarthritis English language Randomized controlled trials, OR prospective uncontrolled trials, OR retrospective cohorts

Exclusion Criteria
Non‐English language Study performed outside of the United States

Abbreviations: BMAC, bone marrow aspirate concentrate; MFAT, microfragmented adipose tissue; PRP, platelet rich plasma.

Results are summarized in Table 3. Of the 25 articles analyzed, 5 (20%) mentioned race,^18^, ^39^, ^43^, ^45^, ^53^ four (16%) reported race in the demographics table,^39^, ^43^, ^45^, ^53^ and one (4%) reported ethnicity and race in the demographics table in one category including “White,” “Black,” and “Hispanic.”^45^ None of the articles reported SES, education level, or income level. In comparison, all articles (25/25, or 100%) reported age, and 23 reported sex/gender (92%).^23^, ^33^, ^34^, ^35^, ^36^, ^37^, ^38^, ^39^, ^41^, ^42^, ^43^, ^44^, ^45^, ^46^, ^47^, ^48^, ^49^, ^50^, ^51^, ^52^, ^53^, ^54^, ^55^

**TABLE 3:** Racial and Ethnic Representation in Studies that Reported Race/Ethnicity.

Study	Racial/Ethnic Representation		
Centeno 2014	White (89.2%)	Non‐White (10.8%)	
	BMC 89.3%	BMC 10.7%	
	BMC + Adipose Graft 88.8%	BMC + Adipose Graft 11.2%	
Shapiro 2017	White 20 (80%)	Non‐White 5 (20%)	
Shapiro 2019	White 20 (80%)	Non‐White 5 (20%)	
Garza 2020	White 32 (82%)	Black 1 (7.7%)	Hispanic 6 (15.4%)
	Placebo 9 (69.2%)	Placebo 1 (7.7%)	Placebo 3 (23.1%)
	Low 12 (92.3%)	Low 0	Low 1 (7.7%)
	High 11 (84.6%)	High 0	High 2 (15.4)
Baria 2022	White 48 (82.8%)	Black 7 (12%)	Other 3 (5.2%)
	PRP 24 (80%)	PRP 4 (13.3%)	PRP 2 (6.7%)
	MFAT 24 (85.7%)	MFAT 3 (10.7)	MFAT 1 (3.6%)

Abbreviations: BMC, bone marrow concentrate; MFAT, microfragmented adipose tissue; PRP, platelet rich plasma.

Of the studies reporting race/ethnicity,^18^, ^39^, ^43^, ^45^, ^53^ most of the studied population were White individuals (80% to 89.2%), followed by Hispanic individuals in one study (15.4%),^45^ and Black individuals as reported in two studies (7.7% to 12%).^45^, ^53^ Three studies did not further characterize “non‐White” individuals, which made up 10.8% to 20% of the studied population.^18^, ^39^, ^43^ None of the studies that were analyzed performed subgroup outcome analysis based on race, ethnicity, other indicators of SES, or social determinants of health (SDOH).

## DISCUSSION

Approximately 10% of the U.S. population has knee osteoarthritis. Previous studies have shown that both non‐White individuals and those with low SES experience higher disease prevalence and disease‐related pain scores.^1^, ^3^, ^6^, ^7^ Although studies evaluating the use of orthobiologics for treating knee osteoarthritis have demonstrated safety and potentially non‐inferior outcomes to alternative non‐operative treatment modalities, the associated “out‐of‐pocket” costs are likely prohibitive. This limitation is likely to restrict widespread access and subsequent inclusion in research. The aim of this review was twofold: first, to elucidate the degree to which study participants from diverse racial/ethnic backgrounds and SES are included in studies investigating the use of orthobiologics as a treatment for knee osteoarthritis; and second, to serve as a call to action to the orthopedic community to advocate for inclusion and reporting of diverse patient populations in research evaluating orthobiologic treatments for knee osteoarthritis to allow for evaluation of the safety and efficacy of these treatments.

Through our literature search, we identified that there is both limited reporting and representation of race/ethnicity, SES, and SDOH for patients with knee osteoarthritis treated with orthobiologics, which limits the ability to evaluate and compare efficacy and safety profiles of orthobiologics as an alternative treatment modality for knee osteoarthritis across populations. Specifically, of the 20% of studies included in our literature search that reported race/ethnicity, 80% or more of all study populations were White. Moreover, no studies reported SES, education level, or income level in relation to study demographics or treatment outcomes. Our current understanding of the safety and efficacy profile of orthobiologics across broad populations may be skewed. Selection bias, stemming from differences between the sample group and target population, may exist due to the limited representation of race/ethnicity, SES, and SDOH among patients with knee osteoarthritis treated with orthobiologics. This bias is likely in part due to the frequent prerequisite for “out‐of‐pocket” payment, which limits use among broader patient populations.

As studies advance our knowledge regarding the safety and efficacy of orthobiologics in treating knee osteoarthritis, it becomes evident that addressing the inadequate representation of diverse racial, ethnic, and SES groups in the literature's coverage of orthobiologics treatment is imperative. Collective solutions from the health care community are essential to rectify these foundational issues. Efforts to ensure equitable inclusion and representation of medical treatments in research, including orthobiologics, must be prioritized to address limitations in the knowledge of safety and efficacy across broad populations. It is widely encouraged in clinical studies to collect demographic and SDOH data to address racial/ethnic disparities in health care.^56^ Aptly, all clinical studies funded by the National Institutes of Health (NIH) are mandated “to ensure the inclusion of … members of racial and ethnic minority groups.^57^” To address the knowledge gap in the literature identified in this review, future studies should incorporate race/ethnicity, SES, and SDOH into orthobiologics study populations.

From a treatment standpoint, approaches to increase access to “out‐of‐pocket” or “cash‐only” procedures (i.e., orthobiologics) involve a combination of strategies tailored to the needs and context of the community. Although applicable to improving access to medical interventions and treatments, Table 4 describes strategies and policies implementable at local, state, and federal levels to increase access to care. A combination of these approaches can be applied and tailored to the needs and dynamics of the community and medical system. Several medical specialties have been confronted with expanding access to elective, “out‐of‐pocket,” or “cash‐only” procedures to patients of broad racial/ethnic and SES classes, including ophthalmologic surgery (e.g., Laser‐Assisted in Situ Keratomileusis [LASIK]), surgical oncology (e.g., postmastectomy breast reconstruction, and cosmetic [aesthetic]) services and surgery (e.g., Botox injections, fillers, and laser hair removal). These specialties utilize a combination of methods outlined in Table 4 to increase access. Similar techniques may also be used when investigating and treating knee osteoarthritis. Given that minority populations and patients with a household income of less than $125,000 per year pursue “out‐of‐pocket” or “cash‐only” procedures regardless of affordability or income status, it would be inferred that there is a demand for safe and effective treatments for knee osteoarthritis among these patient groups.^58^ Moreover, although elective, “out‐of‐pocket,” or “cash‐only” treatments are often not considered essential health care. Safe and effective treatments for knee osteoarthritis should be considered essential and offered equitably to patients in the appropriate clinical context.

**TABLE 4:** Potential strategies and policies that can be used at the local, state, and federal levels to increase access to orthobiologics treatments.

Method	Description	Method	Description
Research	Increase access to care and advance efficacy and safety data across populations	Health Literacy Programs	Educate patients about their condition(s) and treatment options
Insurance	Advocate with insurance companies to cover treatments	Culturally Competent Communication	Provide communication that is apt and culturally sensitive for the target population
Bundle	Offer insurance‐approved bundled care for all treatments related to a specific condition	Financial Support	Offer assistance for treatments to help alleviate financial burden
Mobile Clinics	Conduct mobile clinics in underserved areas; can provide necessary initial screening and referral for advanced treatments	Multilingual Materials	Deliver educational materials in the language(s) spoken by patients to limit language barriers
Transportation Assistance	Contest transportation strains by offering shuttle services or vouchers to health care facilities	Patient Navigators	Assign patients to a patient navigator to assist with understanding, access, and treatments
Payment Plans and Financing	Offer alternatives to extend timeframe of repayment for the cost of treatments	Discounts and Special Offers	Offer campaigns to reduce the overall cost of treatment
Training Programs	Teaching institutions may offer discounted rates for treatments	Partnerships	Partner with local and/or national brands to increase education and/or to provide packages or discounts
Price Transparency	Pricing transparency decreases confusion, particularly for patients with language barriers	Community Outreach	Collaborate with local organizations to educate patients and increase awareness about their condition and treatment options
Tiered Pricing	Vary cost of treatment by household income, complexity of the procedure, etc.	Charitable Programs and Nonprofits	Partner to offer reduced‐cost or free treatment(s)
Insurance Savings Accounts	Increase education about and use of flexible spending accounts and health savings accounts	Military Discounts	Offer discounts to active‐duty or veteran military persons
Policy	Lobby state and federal channels in favor of universal access		

As a narrative review, this study is inherently limited by its study design, which includes the potential constraints in both the quantity and quality of available data, and potentially restricting findings. This review may also offer a biased representation of the use of orthobiologics in mainstream clinical practice due to potential publication biases. Moreover, given the limited data available on the impact of SDOH on the utilization of orthobiologic treatments for knee osteoarthritis in our review, we cannot offer comprehensive, high‐level data on this subject. Furthermore, we acknowledge that we have discussed numerous strategies and policies that can be used to increase access to orthobiologic treatments for patients with knee osteoarthritis, although the applicability and feasibility of these strategies and policies for individual providers, sociocultural conditions, and institutions may vary. Solutions will need to be tailored to local, state, and federal levels to best meet the needs of our patients.

## CONCLUSION

There is emerging evidence suggesting that orthobiologics may be a safe alternative for treating knee osteoarthritis. However, the results of this review revealed that there is a lack of reporting on demographics such as race, ethnicity, SES, and SDOH; and when these are reported that there is a significant underrepresentation of non‐White populations. To address this issue, it is important to purposefully be inclusive in the selection of study populations, and collect and report race, ethnicity, SES, and SDOH.

Inequities significantly contribute to poorer outcomes among patients with knee osteoarthritis. These disparities are exacerbated when payer models restrict access, availability, and funding for orthobiologic treatments. We encourage future studies to address the research gap and evaluate if there is equitable access for all populations to emerging treatment alternatives for knee osteoarthritis.

We hope that this article raises awareness in the orthobiologics community, prompting providers and researchers to address both clinical and research disparities in the use of orthobiologics. Efforts aimed at improving research, education, and community activism are crucial steps toward mitigating these disparities.

## DISCLOSURES

There are no funding sources or conflicts of interest to disclose.
